# Supplementary figures and images for: C. elegans SAS-1 ensures centriole integrity and ciliary function, and operates with SSNA-1
Source: PLoS Genet. 2025 Oct 22;21(10):e1011912. doi: 10.1371/journal.pgen.1011912 (PMC12599939; doi:10.1371/journal.pgen.1011912)

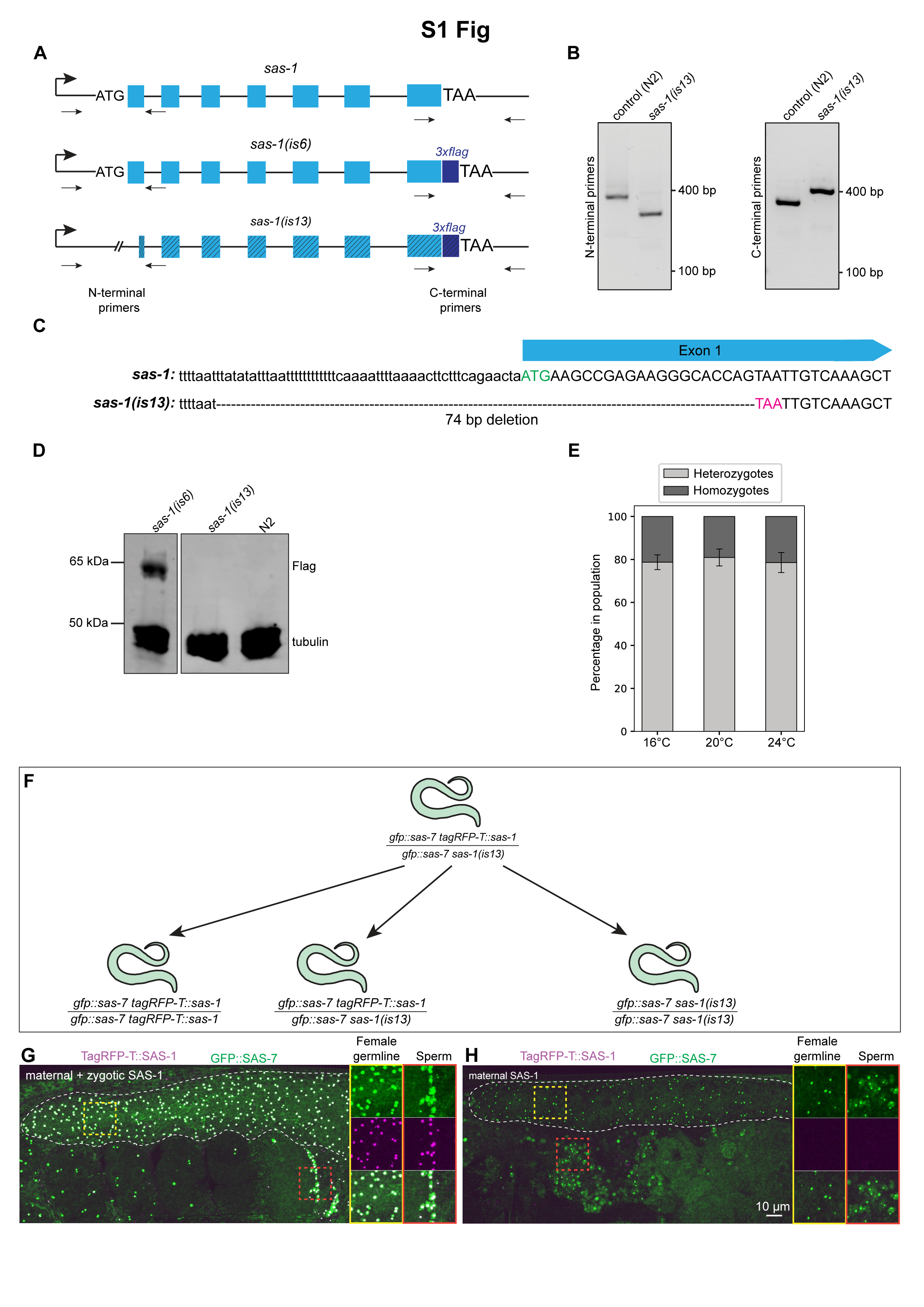

Supplement: S1 Fig — A. Schematic of wild-type sas-1, as well as sas-1(is6) [11], and sas-1(is13) alleles. Primers at the N- and C-terminus used for PCR-based genotyping are indicated. B. Agarose gel image of PCR products from genomic DNA of control and sas-1(is13) mutant worms using primers mentioned in (A). C. Sanger sequencing showing 74 bp deletion in sas-1(is13), stretching from the 5’-UTR to Exon 1, leading to loss of the ATG start codon. D. Western blotting of worm lysates. Note that the ~ 65 kDa band of sas-1::3xflag is lost in sas-1(is13). E. Progeny test of sas-1(is13)/hT2(gfp) worms; N = 8 technical repeats. F. Schematic of genotypes among progeny derived from strain tagRFP-T::sas-1/sas-1(is13) with indication of corresponding animals in S1G and S1H Fig (Created and adapted from BioRender (https://BioRender.com/s7jx4jh)). G, H. Representative live images of gonads of worms expressing GFP::SAS-7 and possibly zygotic or/and maternal TagRFP-T::SAS-1 (G, N = 8 gonads), as well as GFP::SAS-7 and maternal TagRFP-T::SAS-1 only (H, N = 6 gonads). (TIF) [file pgen.1011912.s001.tif]

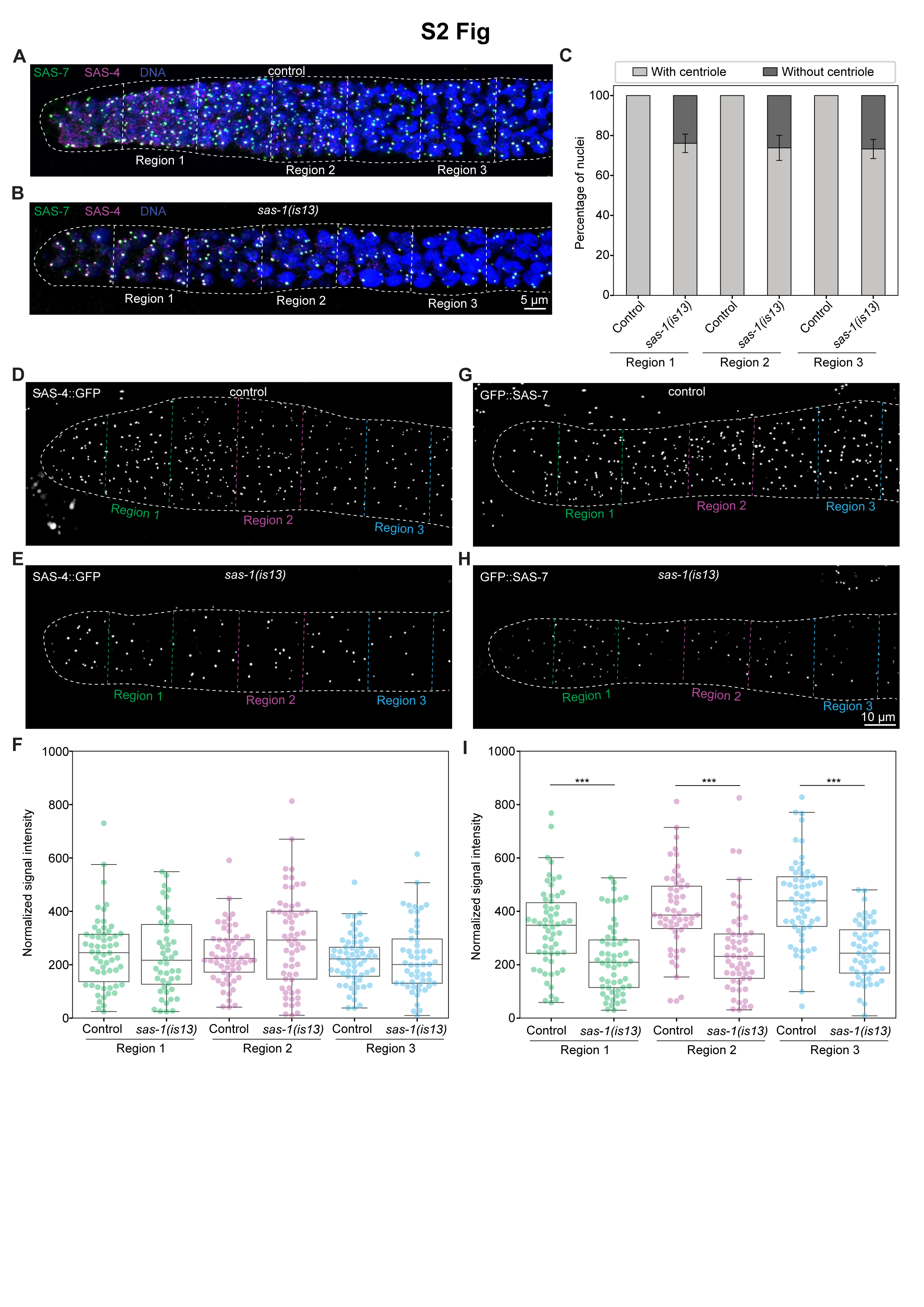

Supplement: S2 Fig — A, B. Representative immunofluorescence images of gonads of control (A) and sas-1(is13) mutant (B), both expressing GFP::SAS-7 and immunostained for GFP and SAS-4. Numbered regions in white mark the positions where signal quantification, shown in C, was performed. C. Quantification of percentage of nuclei with or without centrioles in different regions of distal gonad of control (N = 6 gonads) and sas-1(is13) mutants (5 gonads; N = 230 nuclei for region 1; N = 236 nuclei for region 2; N = 208 nuclei for region 3). D, E, G, H. Live imaging of mitotic region of the gonad in control (D, G) or sas-1(is13) mutant (E, H) worms expressing SAS-4::GFP (D, E) or GFP::SAS-7 (G, H). Three regions of ~18 µm in width and ~18 µm apart were defined as indicated. Signal intensity of SAS-4::GFP and GFP::SAS-7 was then quantified in these regions. F, I. Quantification of signal intensity of SAS-4::GFP (F) and GFP::SAS-7 (I) in the three regions described in (D, E, G, H). Foci number for F: control (5 gonads; N = 58 for region 1, 61 for region 2, 61 for region 3), sas-1(is13) (5 gonads; N = 48 for region 1, 60 for region 2, 54 for region 3). Foci number for I: control (5 gonads; N = 58 for region 1, 55 for region 2, 59 for region 3), sas-1(is13) (5 gonads, N = 56 for region 1, 56 for region 2, 51 for region 3). Student’s two-tailed t-tests, whereby P < 0.001 (***). The differences between control and sas-1(is13) in F are not significant (Region 1: P = 0.77, Region 2: P = 0.055, Region 3: P = 0.69). (TIF) [file pgen.1011912.s002.tif]

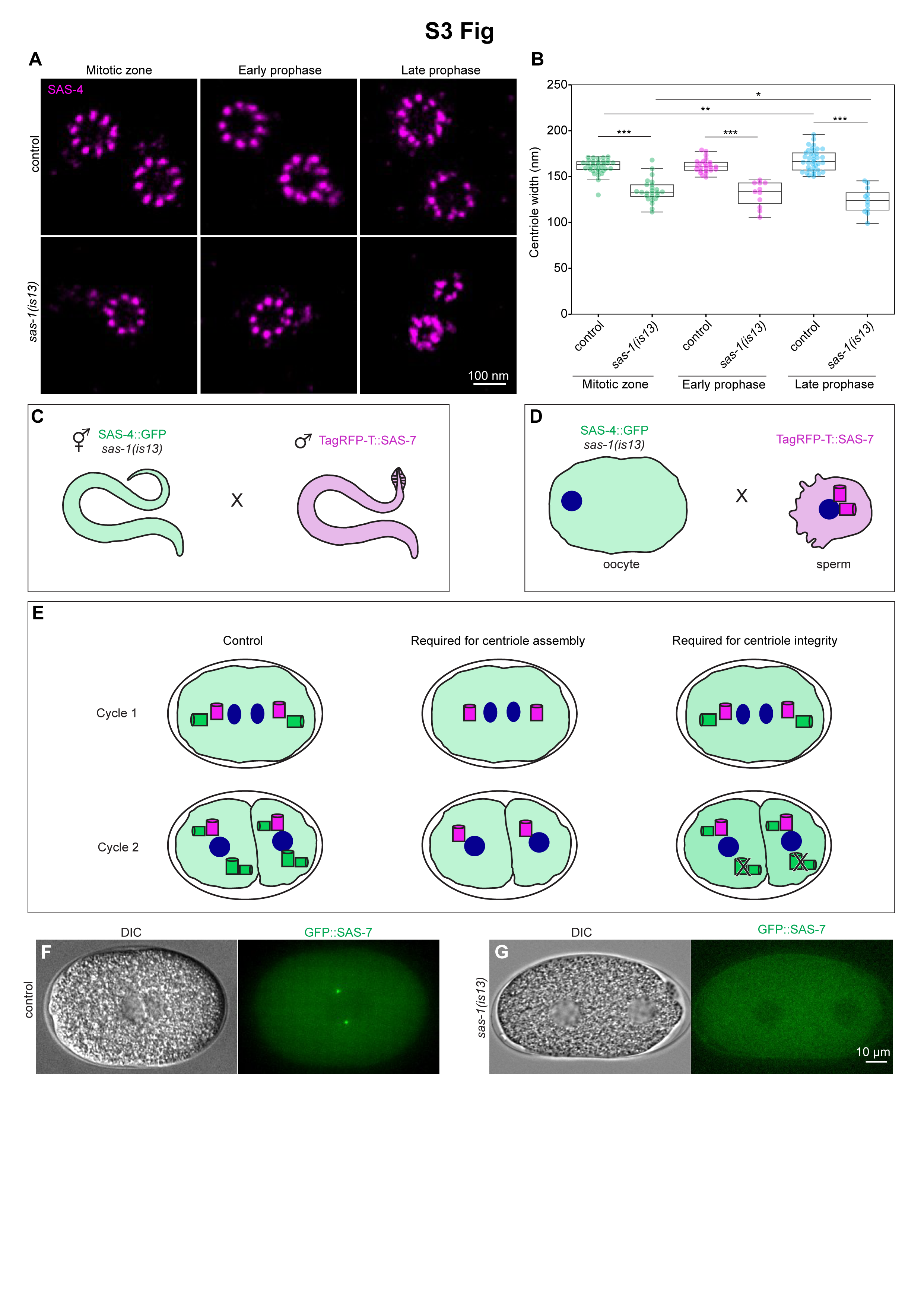

Supplement: S3 Fig — A. Representative U-Ex-STED images of centrioles immunostained for SAS-4 in the mitotic zone, early prophase, and late prophase of control (N = 15, 14 and 20, respectively) and sas-1(is13) mutant (N = 18, 10 and 10, respectively) gonads. Scale bar is corrected for the expansion factor of 5. B. Quantification of centriole width in indicated regions of control and sas-1(is13) mutant gonads from A. Student’s two-tailed t-tests, whereby P < 0.05 (*); P < 0.01 (**); P < 0.001 (***). C, D. sas-1(is13) mutant hermaphrodites expressing SAS-4::GFP (green) mated with control males expressing TagRFP-T::SAS-7 (magenta). (Created and adapted from BioRender (https://BioRender.com/s7jx4jh) as well as [63]). E. Possible outcomes of marked mating experiment: (left) Control; in this case, in cycle 2, two SAS-4::GFP foci are present in both blastomeres, which undergo bipolar spindle assembly. (middle) SAS-1 is essential for centriole assembly; in this case, no SAS-4::GFP foci are present in the embryo, and monopolar spindle assembly occurs in both blastomeres at the two-cell stage. (right) SAS-1 is essential for centriole integrity; in this case, two SAS-4::GFP foci are present in each blastomere initially but are eliminated thereafter (indicated by black cross). F, G. Snapshots from live imaging at the time of pronuclear meeting in control (F; N = 10) and sas-1(is13) mutant (G; N = 9) one-cell stage embryos. Images were acquired every minute. The DIC channel is a single plane, whereas the GFP::SAS-7 channel is a maximum intensity projection of selected Z-planes. Whereas both sperm-contributed centrioles are invariably visible at pronuclear meeting in the control (N = 10), no focus of GFP::SAS-7 is present at the equivalent stage in sas-1(is13) mutant embryos (N = 9), although one was detected at earlier stages in some embryos (3/9). (TIF) [file pgen.1011912.s003.tif]

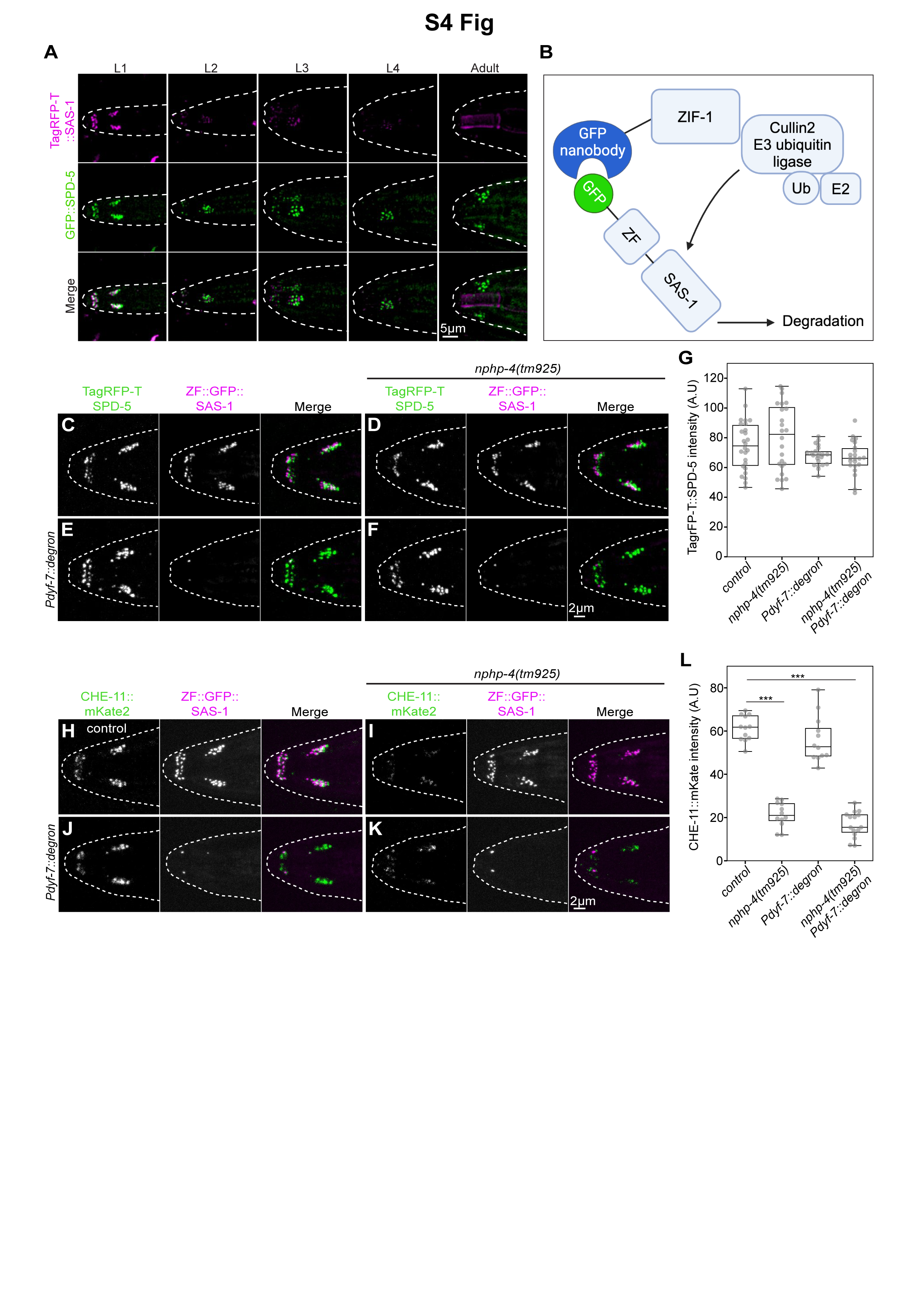

Supplement: S4 Fig — A. Live imaging of sensory cilia at anterior of animals expressing TagRFP-T::SAS-1 and GFP::SPD-5 during larval stages and adulthood, as indicated. N = 10 for each stage. The bright cylindrical signal observed in the TagRFP-T channel in the adult stage is likely autofluorescence from the mouth orifice. B. Schematic of ZIF-1 mediated degron system, with ZIF-1 expression under the control of the dyf-7 promoter in this case. C-F. Live imaging of L1 larvae expressing TagRFP-T::SPD-5 and ZF::GFP::SAS-1 in control (C), nphp-4(tm925) (D), Pdyf-7::degron (E, note two remaining ZF::GFP::SAS-1 foci, likely due to lack of ZIF-1 expression in these neurons), and Pdyf-7::degron nphp-4(tm925) (F) animals. G. Quantification of TagRFP-T::SPD-5 intensity in S4C–S4F Fig. Rectangular ROI of 15.82 µm2 was drawn around amphid cilia or adjacent worm body (for background subtraction) in the sum projected Z-slices of 6 µm. TagRFP-T::SPD-5 signal reported is the background subtracted mean signal intensity of amphid cilia. N = 26 (control, from 13 animals); N = 24 (nphp-4(tm925), from 12 animals); N = 22 (Pdyf-7::degron, from 11 animals), N = 22 (Pdyf-7::degron nphp-4(tm925), from 11 animals). Student’s two-tailed t-tests, which were all not significant compared to control; nphp-4(tm925): P = 0.34, Pdyf-7::degron: P = 0.07, Pdyf-7::degron nphp-4(tm925), P = 0.05. H-K. Live imaging of L1 larvae expressing CHE-11::mKate2 and ZF::GFP::SAS-1 in control (H), nphp-4(tm925) (I), Pdyf-7::degron (J), and Pdyf-7::degron nphp-4(tm925) (K) animals. L. Quantification of CHE-11::mKate2 intensity in S4H–S4K Fig. Quantification was performed as mentioned in S4G Fig. N = 12 (control, from 6 animals); N = 12 (nphp-4(tm925), from 6 animals); N = 12 (Pdyf-7::degron, from 6 animals), N = 15 (Pdyf-7::degron nphp-4(tm925), from 8 animals). Student’s two-tailed t-tests and comparison was made with control; nphp-4(tm925): P <0.001 (***), Pdyf-7::degron: P = 0.16, Pdyf-7::degron nphp-4(tm925), P <0.001 (***). Compari [file pgen.1011912.s004.tif]

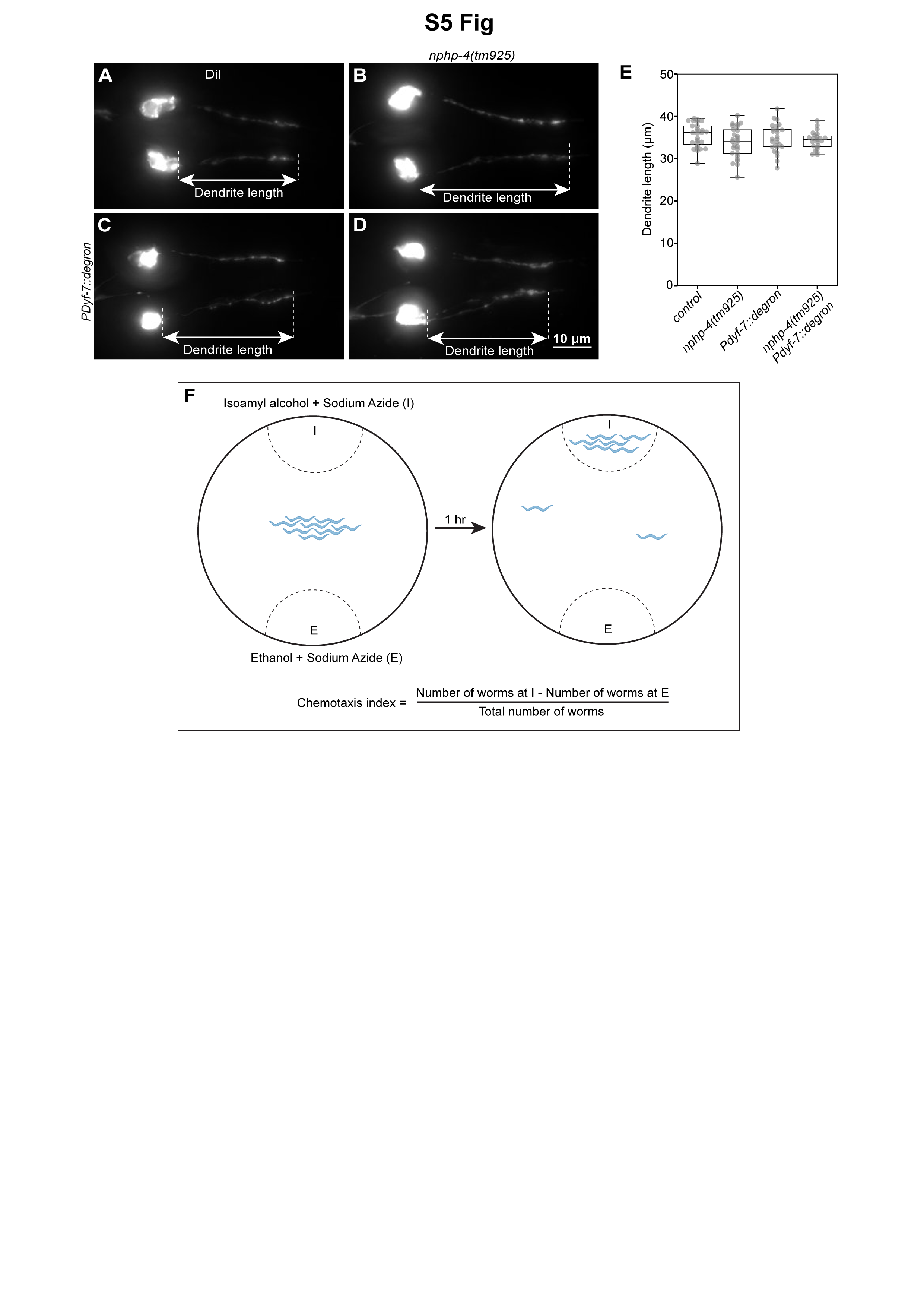

Supplement: S5 Fig — A-D. Representative image of phasmid neurons stained with DiI in control (A), nphp-4(tm925) (B), Pdyf-7::degron (C), Pdyf-7::degron nphp-4(tm925) (D) animals. Dendrite length was measured as illustrated by drawing a segmented line from the end of the neuron cell body until the beginning of cilium (which stains much more faintly than rest of the neuron by DiI and can hence be identified easily). Images are maximum intensity projections of selected Z-planes. E. Quantification of dendrite length from A-D. Dendrites quantified: N = 27 (control, from 14 animals); N = 27 (nphp-4(tm925), from 14 animals); N = 26 (Pdyf-7::degron, from 14 animals), N = 23 (Pdyf-7::degron nphp-4(tm925), from 12 animals). Student’s two-tailed t-tests, which were all not significant compared to control vs nphp-4(tm925): P = 0.06, Pdyf-7::degron: P = 0.4, Pdyf-7::degron nphp-4(tm925), P = 0.09. F. Schematic of chemosensation assay (not to scale) (Created and adapted from BioRender (https://BioRender.com/8ba9yul)). See text for further details. (TIF) [file pgen.1011912.s005.tif]

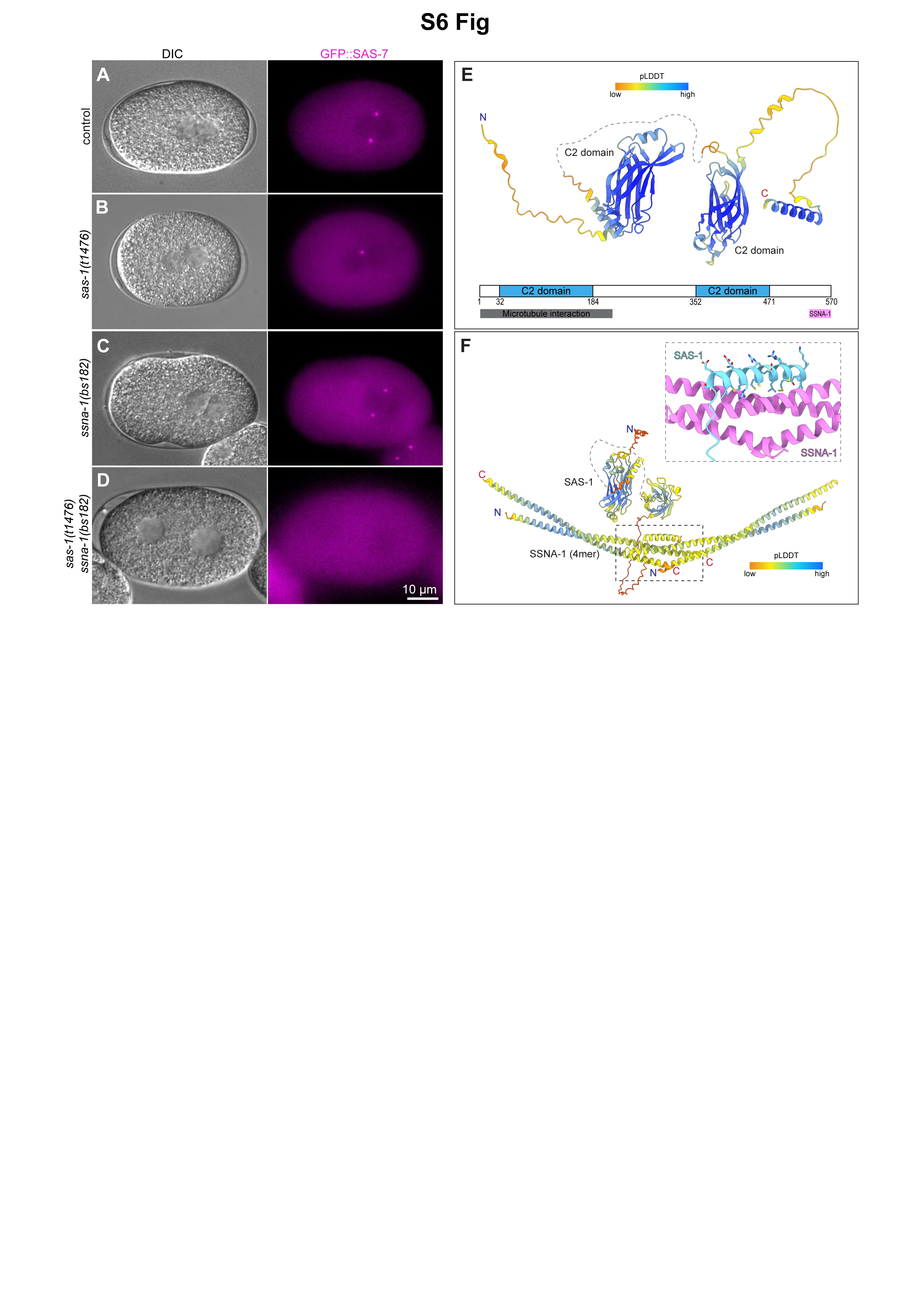

Supplement: S6 Fig — A-D. Snapshots from time-lapse imaging showing DIC and GFP::SAS-7 distribution in embryos at pronuclear meeting in control (A; N = 10), sas-1(t1476) (B; N = 10), ssna-1(bs182) (C, N = 10), and sas-1(t1476); ssna-1(bs182) (D; N = 7) embryos. DIC channel is a single plane whereas the GFP::SAS-7 channel is a maximum intensity projection of selected Z-planes. E. AlphaFold2 prediction of SAS-1 with two C2-domains and a C-terminal alpha helix. The flexible loop connecting the two C2 domains is indicated with dotted line. F. AlphaFold2 prediction of SAS-1 binding to SSNA-1 tetramer. SSNA-1 is forming a head-to-tail fibril with the C-terminal helix of SAS-1 binding to the interface of two helixes. Top-right: magnified representation of the interface marked with dotted line; SAS-1 in cyan, SSNA-1 in pink. (TIF) [file pgen.1011912.s006.tif]

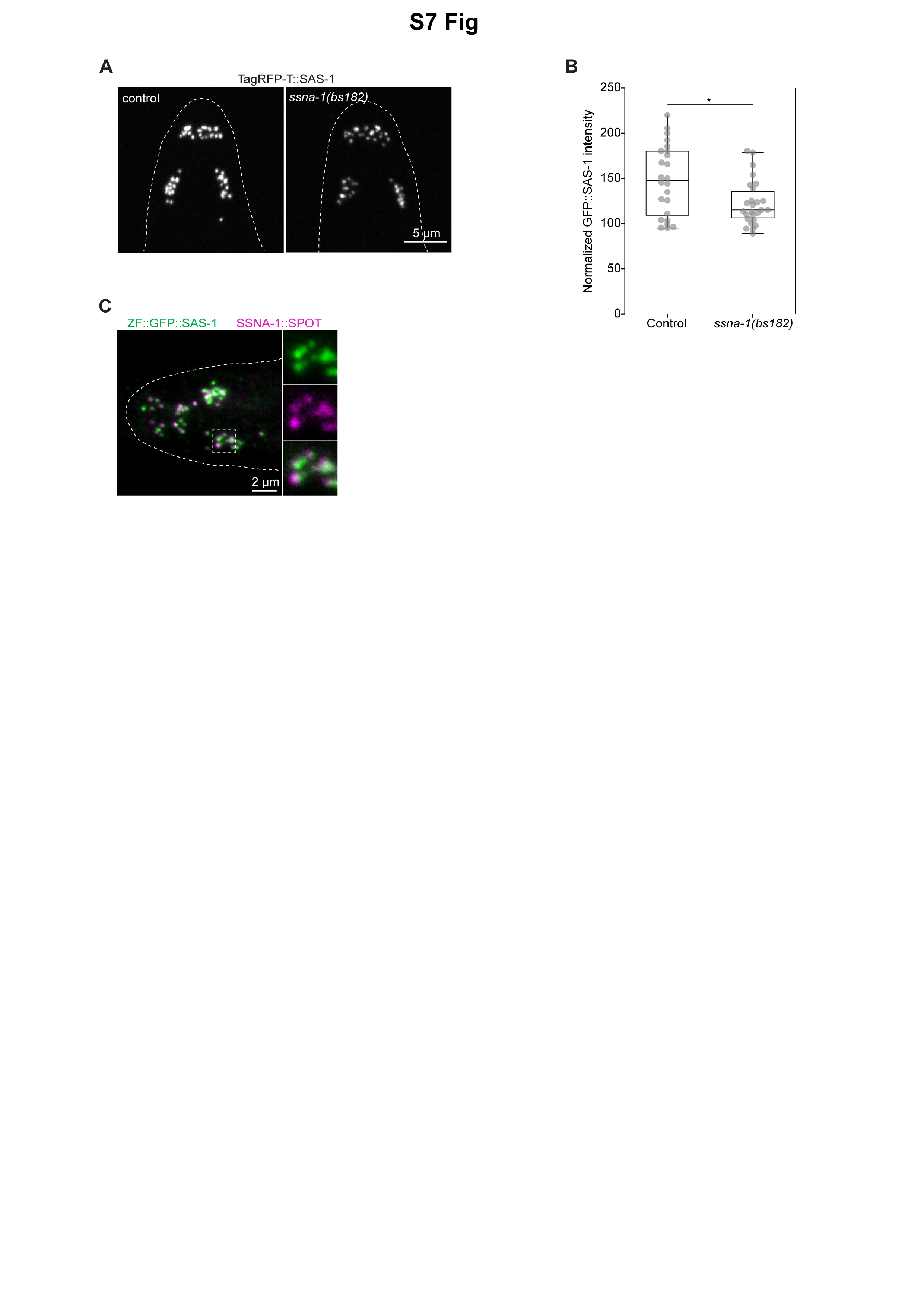

Supplement: S7 Fig — A. Live imaging of anterior cilia of L1 larvae expressing TagRFP-T::SAS-1 in control (N = 12) and ssna-1(bs182) mutant (N = 13). B. Quantification of TagRFP-T::SAS-1 signal intensity in anterior sensory cilia from S7A. Quantification was performed as mentioned in S4G Fig with a ROI of ~12 µm2. N = 24 from 12 control worms, and N = 26 from 13 ssna-1(bs182) mutants. Student’s two-tailed t-tests, whereby P < 0.05 (*). C. Representative immunofluorescence image of L1 larva stained for ZF:GFP::SAS-1 (green) and SSNA-1::SPOT (magenta). Insets are 2 times magnified. (TIF) [file pgen.1011912.s007.tif]

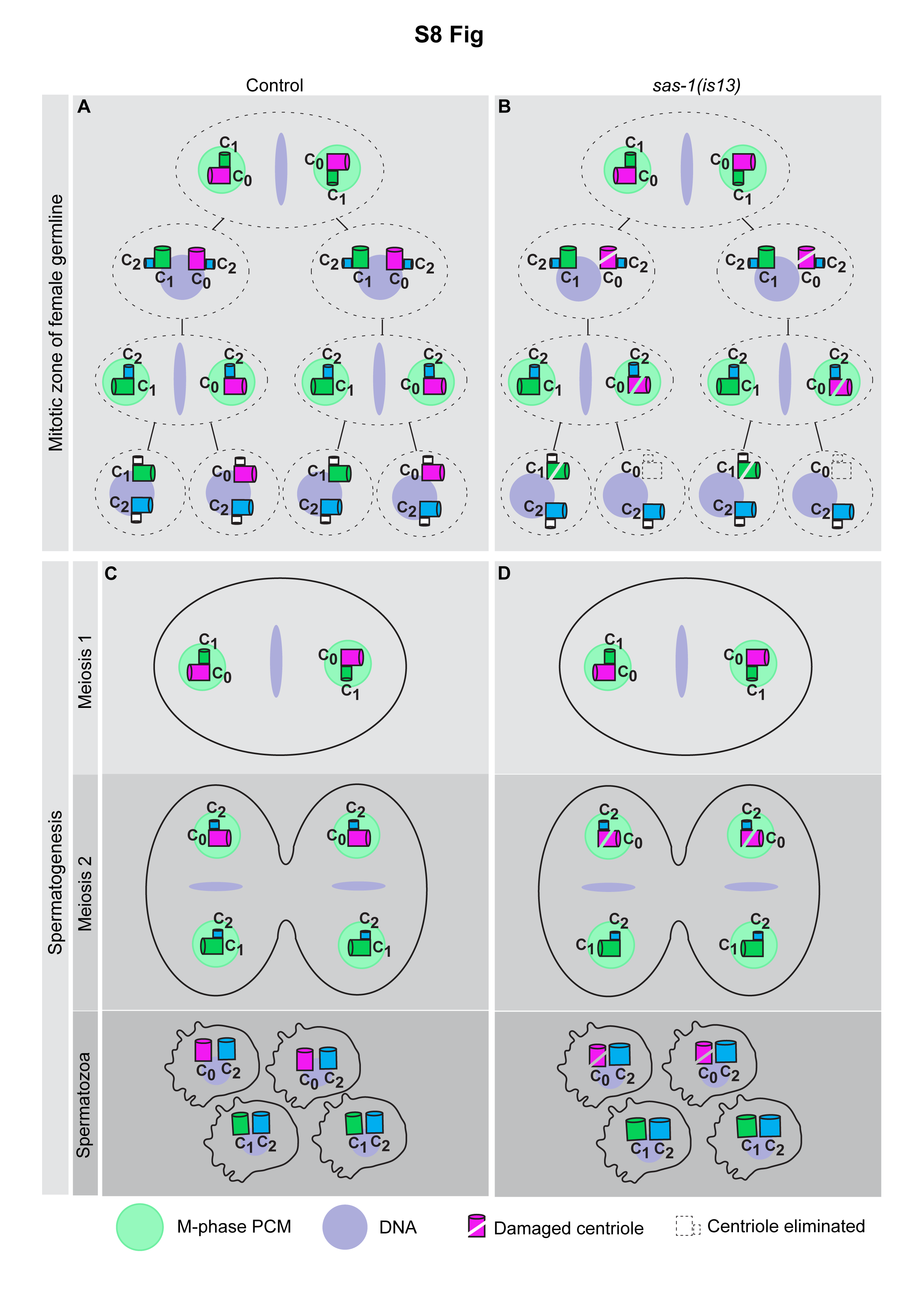

Supplement: S8 Fig — A, B. Schematic of two rounds of centriole duplication in the mitotic region of the female germline in control (A) and sas-1(is13) mutant (B) animals. Dashed lines indicate cytoplasmic area around individual germ line nuclei. In the control, the two pre-existing centrioles (C0, magenta) each gave rise to one new centriole (C1, green), which matured during mitosis (top row). In the following interphase, all four centrioles (2x C0 and 2x C1) mentor assembly of one new centriole each (C2, blue) (second row). In the following mitosis, C2 centrioles mature (third row). In the next interphase (last row), all centrioles mentor assembly of one new centriole each (white). In sas-1(is13) mutants, centrioles are generated, but lose structural integrity as they traverse the subsequent mitoses. As a result, germ cell nuclei at the end of the second mitosis exhibit two types of configurations. In both types, the first, younger, centriole (C2) is intact, whereas the second, older, centriole is either deformed (C1) or absent (C0), depending whether it was formed in the previous cell cycle (C1) or earlier than that (C0). Note also that ~20% of sas-1(is13) nuclei are devoid of centrioles (see S2C Fig), so that organelle loss must be more drastic in some cases, perhaps in those configurations with the oldest C0 centrioles. C, D. Schematic of centriole duplication during the meiotic divisions of spermatogenesis in control (C) and sas-1(is13) mutant (D) animals. In the control, the two pre-existing centrioles (C0, magenta) each gave rise to one new centriole prior to the first meiotic division (C1, green). All four centrioles (2x C0 and 2x C1) mentor assembly of one new centriole each (C2, blue) prior to the second meiotic division. In sas-1(is13) mutants, centrioles are assembled as in the control, but the two that were present initially (C0) lose structural integrity during the two meiotic divisions, so that they are ruptured in mature sperm. As a consequence, sperm exhibit two config [file pgen.1011912.s008.tif]
