# Supplementary material for: C. elegans SAS-1 ensures centriole integrity and ciliary function, and operates with SSNA-1
Source: PLoS Genet. 2025 Oct 22;21(10):e1011912. doi: 10.1371/journal.pgen.1011912 (PMC12599939; doi:10.1371/journal.pgen.1011912)
Supplement: S1 Table — The table provides the complete list of C. elegans strains used in this study. (PDF) [file pgen.1011912.s010.pdf]

Table S1: list of strains used in this study

Lab designation	Genotype	References
GZ1385	*sas-7(or1940[gfp::sas-7])III; glo-1(zu931)X*	[1]
GZ2134	*sas-7(or1940[gfp::sas-7])III; sas-6(ok2554)IV/nT1(qIs51)IV; glo-1(zu931)X*	[1,2]; this study
GZ2122	*sas-7(or1940[gfp::sas-7])III sas-1(is13)III/hT2; ssna-1(bs206 [ssna-1::spot]) IV*	[1,3]; this study
GZ1816	*sas-4(bs195[sas-4::gfp] III; glo-1(zu931)X*	[4]
GZ2140	*sas-4(bs195[sas-4::gfp] III sas-1(is13)III/hT2; ssna-1(bs206 [ssna-1::spot]) IV*	[3,4]; this study
GZ2123	*zif-1(gk117) III sas-7(is1[tagRFP::sas-7+loxP])III sas-1(is12[zf::gfp:sas-1]III); ssna-1(bs206 [ssna-1::spot]) IV*	[3,5]; this study
GZ1754	*stIs10544 [hlh-16::H1-wCherry::let-858 3' UTR], sas-7(or1940[gfp::sas-7])III, glo-1(zu931)X*	[4]
GZ2135	*stIs10544 [hlh-16::H1-wCherry::let-858 3' UTR], zif-1(gk117) III sas-7(is1[tagRFP::sas-7+loxP])III sas-1(is12[zf::gfp:sas-1]III)*	[4]; this study
GZ2050	*sas-7(or1940[gfp::sas-7])III sas-1(is10[TagRFP-T::sas-1])III; glo-1(zu931)X*	[1]; this study
GZ2071	*spd-5(vie26[gfp::spd-5 +loxP])I; sas-1(is10[TagRFP-T::sas-1])III*	[6]; this study
GZ2118	*vieSi22[pAD401; Pmksr-2::gfp::mksr-2; cb unc-119(+)]I; sas-1(is10[TagRFP-T::sas-1])III; glo-1(zu931)X*	[7]; this study
GZ2115	*zif-1(gk117)III sas-7(is1[tagRFP::sas-7+loxP])III sas-1(is12[zf::gfp:sas-1]III); vieSi70[pAD675;pche-11::che-11::mKate2] IV*	[5,8]; this study

GZ2106	*ltSi1017[pDC335; Pdyf-7::vhhgfp4::ZIF-1;cb-unc-119(+)] II; unc-119(ed3) III? zif-1(gk117)III sas-7(is1[tagRFP::sas-7+loxP])III sas-1(is12[zf::gfp:sas-1]III); vieSi70[pAD675;pche-11::che-11::mKate2] IV*	[5,8]; this study
GZ2119	*zif-1(gk117)III sas-7(is1[tagRFP::sas-7+loxP])III sas-1(is12[zf::gfp:sas-1]III); vieSi70[pAD675;pche-11::che-11::mKate2] IV; nphp-4(tm925)V*	[5,7,8]; this study
GZ2120	*ltSi1017[pDC335; Pdyf-7::vhhgfp4::ZIF-1;cb-unc-119(+)] II; unc-119(ed3) III? zif-1(gk117)III sas-7(is1[tagRFP::sas-7+loxP])III sas-1(is12[zf::gfp:sas-1]III); vieSi70[pAD675;pche-11::che-11::mKate2] IV; nphp-4(tm925)V*	[5,8]; this study
GZ2116	*sas-7(or1940[gfp::sas-7])III sas-1(is10[TagRFP-T::sas-1])III; ssna-1(bs182)/ears-2(ve631[LoxP + myo-2p::GFP::unc-54 3' UTR + rps-27p::neoR::unc-54 3' UTR + LoxP]) IV*	[1,3]; this study
GZ2080	*sas-1(is7[3xflag::sas-1])III; ssna-1(bs206 [ssna-1::spot]) IV*	[3,9]
GZ1914	*sas-7(or1940[gfp::sas-7])III, sas-1(t1476)/hT2; glo-1(zu931)X*	[1,10]; this study
GZ2141	*sas-7(or1940[gfp::sas-7])III sas-1(t1476)/hT2 III; ssna-1(bs182)/ears-2(ve631[LoxP + myo-2p::GFP::unc-54 3' UTR + rps-27p::neoR::unc-54 3' UTR + LoxP]) IV*	[1,3,10]; this study
GZ2076	*sas-7(or1940[gfp::sas-7])III sas-1(is10[TagRFP-T::sas-1])III; ssna-1(bs182)/ears-2(ve631[LoxP + myo-2p::GFP::unc-54 3' UTR + rps-27p::neoR::unc-54 3' UTR + LoxP]) IV*	[1,3,10]; this study

GZ2136	*ltSi1017[pDC335; Pdyf-7::vhhgfp4::ZIF-1;cb-unc-119(+)] II; unc-119(ed3) III? zif-1(gk117)III sas-7(is1[tagRFP::sas-7+loxP])III sas-1(is12[zf::gfp:sas-1]III); ssna-1(bs206 [ssna-1::spot]) IV*	[3,5,8]; this study
GZ2151	*spd-5(wow36[tagrfp-t^3xmyc::spd-5])I; ltSi1017[pDC335; Pdyf-7::vhhgfp4::ZIF-1;cb-unc-119(+)] II; unc-119(ed3) III? zif-1(gk117)III sas-7(is1[tagRFP::sas-7+loxP])III sas-1(is12[zf::gfp:sas-1]III); nphp-4(tm925)V*	[5,8,11]; this study
GZ2152	*spd-5(wow36[tagrfp-t^3xmyc::spd-5])I; ltSi1017[pDC335; Pdyf-7::vhhgfp4::ZIF-1;cb-unc-119(+)] II; unc-119(ed3) III? zif-1(gk117)III sas-7(is1[tagRFP::sas-7+loxP])III sas-1(is12[zf::gfp:sas-1]III)*	[5,8,11]; this study
GZ2153	*spd-5(wow36[tagrfp-t^3xmyc::spd-5])I; unc-119(ed3) III? zif-1(gk117)III sas-7(is1[tagRFP::sas-7+loxP])III sas-1(is12[zf::gfp:sas-1]III)*	[5,8,11]; this study
GZ2154	*spd-5(wow36[tagrfp-t^3xmyc::spd-5])I; unc-119(ed3) III? zif-1(gk117)III sas-7(is1[tagRFP::sas-7+loxP])III sas-1(is12[zf::gfp:sas-1]III); nphp-4(tm925)V*	[5,8,11]; this study

Reference

1. Sugioka K, Hamill DR, Lowry JB, McNeely ME, Enrick M, Richter AC, et al. Centriolar SAS-7 acts upstream of SPD-2 to regulate centriole assembly and pericentriolar material formation. eLife. 2017;6: e20353. doi:10.7554/eLife.20353

2. Kitagawa D, Flückiger I, Polanowska J, Keller D, Reboul J, Gönczy P. PP2A Phosphatase Acts upon SAS-5 to Ensure Centriole Formation in C. elegans Embryos. Developmental Cell. 2011;20: 550–562. doi:10.1016/j.devcel.2011.02.005

3. Pfister JA, Agostini L, Bournonville L, Perrier A, Sankaralingam P, Bell ZG, et al. C. elegans SSNA-1 is required for the structural integrity of centrioles and bipolar spindle assembly. Nature Communications. 2025;16: 5220. doi:10.1038/s41467-025-59939-0

4. Kalbfuss N, Gönczy P. Extensive programmed centriole elimination unveiled in *C. elegans* embryos. Sci Adv. 2023;9: eadg8682. doi:10.1126/sciadv.adg8682

5. Klinkert K, Levernier N, Gross P, Gentili C, Von Tobel L, Pierron M, et al. Aurora A depletion reveals centrosome-independent polarization mechanism in Caenorhabditis elegans. eLife. 2019;8: e44552. doi:10.7554/eLife.44552

6. Pierron M, Woglar A, Busso C, Jha K, Mikeladze-Dvali T, Croisier M, et al. Centriole elimination during *Caenorhabditis elegans* oogenesis initiates with loss of the central tube protein SAS-1. The EMBO Journal. 2023;42: e115076. doi:10.15252/embj.2023115076

7. Schouteden C, Serwas D, Palfy M, Dammermann A. The ciliary transition zone functions in cell adhesion but is dispensable for axoneme assembly in *C. elegans*. Journal of Cell Biology. 2015;210: 35–44. doi:10.1083/jcb.201501013

8. Garbrecht J, Laos T, Holzer E, Dillinger M, Dammermann A. An acentriolar centrosome at the C. elegans ciliary base. Current Biology. 2021;31: 2418-2428.e8. doi:10.1016/j.cub.2021.03.023

9. Woglar A, Pierron M, Schneider FZ, Jha K, Busso C, Gönczy P. Molecular architecture of the C. elegans centriole. Basto R, editor. PLoS Biol. 2022;20: e3001784. doi:10.1371/journal.pbio.3001784

10. Von Tobel L, Mikeladze-Dvali T, Delattre M, Balestra FR, Blanchoud S, Finger S, et al. SAS-1 Is a C2 Domain Protein Critical for Centriole Integrity in C. elegans. Dutcher SK, editor. PLoS Genet. 2014;10: e1004777. doi:10.1371/journal.pgen.1004777

11. Magescas J, Zonka JC, Feldman JL. A two-step mechanism for the inactivation of microtubule organizing center function at the centrosome. eLife. 2019;8: e47867. doi:10.7554/eLife.47867
