# Supplementary material for: C. elegans SAS-1 ensures centriole integrity and ciliary function, and operates with SSNA-1
Source: PLoS Genet. 2025 Oct 22;21(10):e1011912. doi: 10.1371/journal.pgen.1011912 (PMC12599939; doi:10.1371/journal.pgen.1011912)
Supplement: Attachment — Submitted filename: 157252_1_rebuttal_3404877_sx1966.pdf [file pgen.1011912.s012.pdf]

Revision Plan

Review COMMONS

Manuscript number: RC-2025-02998
Corresponding author(s): Pierre Gönczy

1. General Statements [optional]

We thank the three reviewers for their overall appreciation of our work, as well as for inviting us to resolve a few points, including some through additional experiments. We delineate hereafter the steps that we will take to address in full the comments made by the reviewers.

2. Description of the planned revisions

Reviewer #1
Evidence, reproducibility and clarity
SAS-1, a homolog to human C2CD3, is known to contribute to centriole integrity in C. elegans, though the molecular basis of this function has remained elusive. Here, Jha et al. generate the sas-1 null allele and employ U-Ex-STED microscopy to show that while SAS-1 is dispensable for centriole assembly, it is critical for maintaining organelle integrity. The authors also demonstrate that SAS-1 localizes to the transition zone of sensory neurons in L1 larvae. Using the ZIF-1-mediated tissue-specific degradation system in combination with an nphp-4 null mutant, a Y-link transition zone protein, they show that SAS-1 depletion exacerbates the posterior cilia shortening and chemotaxis defects observed in the null nphp-4 mutant, suggesting a partially redundant role for SAS-1 in ciliary function. Additionally, they demonstrate that SSNA-1, the C. elegans Sjögren Syndrome Nuclear Antigen 1 protein, localizes close to the SAS-1 C-terminus in the centriole architecture. SSNA-1 localization to the centriole during oogenesis and to the transition zone during ciliogenesis depends on SAS-1. Moreover, in the human co-expression system, SSNA-1 is recruited to the microtubule in a SAS-1-dependent manner. Collectively, these findings suggest that SAS-1 collaborates with SSNA-1 to maintain centriole integrity and support proper ciliary function.

Major Points
1. The title of the paragraph on Page 6, Line 2, "Loss of SAS-1 leads to premature centriole elimination during oogenesis," is misleading, as the authors do not present direct observation of centriole elimination in the germline. In wildtype, centriole elimination occurs from late pachytene to diplotene stage (PMID: 37987153). To support the current claim, it is necessary to examine SAS-4 and/or SAS-7 signals in the germline of control and sas-1(is13), to investigate whether the loss of SAS-1 leads to premature departure of SAS-4 and/or SAS-7 during meiotic prophase. Otherwise, I recommend rephrasing the title to more accurately reflect the data presented.
> We thank the reviewer for bringing up this important point. Accordingly, we will examine the architecture of centrioles in control and *sas-1(is13)* mutant animals during meiotic prophase using antibodies against SAS-4 and tubulin, and potentially rephrase the section title as a result. See also point 1 of reviewer #3.

2. The localization of SAS-1 should be examined in Figure 1B-D and/or Figure 1G-I. Although protein expression of SAS-1 in wildtype and sas-1(is13) was assessed by Western blotting in Figure S1D, this

analysis was performed on whole worm extracts. Therefore, it remains possible that residual SAS-1 protein is still present in the mitotic region, as the authors themselves note on page 7, lines 22-25. To address this concern, direct visualization of SAS-1 localization in the mitotic region of the gonad is necessary.
> We thank the reviewer for this excellent suggestion. We will test whether SAS-1 provided maternally by animals of genotype *sas-1*(*is13*)/+ perdures in the germline of homozygous *sas-1*(*is13*) mutant adults.

3. There is no explanation provided for the difference between Figures 2C and 2D, nor for the percentages shown in the top-right corners of the merged image in each condition.
> We apologize for not having been sufficiently clear here, and will provide the required explanation in the figure legend.

4. It is necessary to include SAS-7 localization in sperm centriole in Figure 2B-D, or alternatively, SAS-4 localization in Figure 2G-H. This inconsistent use of centriole markers makes it difficult to support the conclusion that the loss of SAS-1 allows sperm centrioles to be assembled but compromises their integrity, presumably leading to their rapid demise in the embryo following fertilization.
> Prompted by the reviewer's suggestion, we will repeat these experiments in order to report the same marker in sperm and embryo centrioles, thereby allowing for a more coherent comparison between conditions.

5. In Figure 4L-O, it is unclear how the authors defined the cilia length. For example, in Figure 4M, the distribution of CHE-11::mKate2 appears longer than the length indicated by the arrow. It would be helpful to provide a clear explanation of how cilia length was measured and to consider using an additional marker, such as OSM-6::GFP (PMID: 18316409), to more accurately define cilia length.
> We thank the reviewer for suggesting to clarify how we define cilium length. We will improve the rendition of Figure 4K-P to clarify this point. Unfortunately, OSM-6::GFP is not compatible with ZF::GFP::SAS-1 (as both proteins are fused to GFP), so that we will use SPY-650-tubulin instead (as reported in Figure 4F), which reliably marks axonemal microtubules.

6. There is no direct evidence demonstrating that the C-terminus of SAS-1 binds to SSNA-1. In the human expression system shown in Figure 6, it would be helpful to test whether a C-terminal deletion of SAS-1 fails to recruit SSNA-1 to the microtubules. This would strengthen the conclusion regarding their interaction.
> Prompted by this suggestion, we will test whether a variant of SAS-1 lacking the C-terminal region recruits SSNA-1 to microtubules in U2-OS cells.

Minor Points
1. In Figure 2A, the position of inset 3 at the 9 min timepoint appears misaligned relative to the corresponding DIC image.
> Thank you for noticing this misalignment, which will be corrected.

2. On page 10, line 34, the authors state that "ZIF-mediated depletion of SAS-1 from cilia results in a slight but significant chemosensation defect, which is enhanced in the nphp-4(tm925) background." However, in Figure 4R, no statistical test (e.g., t-tests) is shown comparing the control and Pdy-

7::degron, and the data do not appear to show a clear difference between these two groups. I recommend rephrasing this conclusion.
> We will conduct statistical analysis of the data shown in Figure 4R and alter the text accordingly in the revised manuscript.

3. In Figure 5C, it is unclear why SAS-1 and SSNA-1 are examined at centrioles during meiotic prophase rather than in early embryos. The shift in experimental system without explanation makes this part of the manuscript difficult to interpret. It would be helpful to explain why meiotic prophase was chosen for this analysis.
> We chose meiotic prophase for U-Ex-STED here because the relatively thin and well-spread gonad tissue allows for higher spatial resolution compared to early embryos, where tissue thickness limits imaging quality. We will clarify this rationale in the revised manuscript.

4. It is unclear why sperm can assemble but then disassemble after fertilization (as shown in Figure 2B-H). Addressing this point in the discussion would help clarify the underlying mechanisms and better contextualize the findings.
> Prompted by the reviewer's request, we will address this point in the discussion of the revised manuscript, considering potential mechanisms that may explain why centrioles contributed by *sas-1(is13)* mutant sperm disassemble shortly after fertilization.

Significance
This manuscript presents significant and new mechanistic insights for the field, demonstrating that SAS-1 ensures centriole integrity and contributes to proper ciliary function through its collaboration with SSNA-1. These findings are supported by the precise generation of a sas-1 null mutant and the establishment of a neuron-specific degradation system. However, several conclusions appear overinterpreted, and the inconsistent use of centriole makers and tissue localization, as well as insufficient explanations of experimental design, make the manuscript difficult to interpret. Therefore, further validation is needed, along with clearer explanations of the experimental systems and quantification methods, as well as rephrasing, as described in major and minor points.

Reviewer #2
Evidence, reproducibility and clarity
This study investigates the role of the centriolar protein SAS-1 in centriole integrity, disassembly, and formation of functional sensory neurons. Previous studies already used a loss of function alleles for sas-1 to demonstrate that SAS-1 affects centriole disassembly and integrity in the gonad of C. elegans. This paper makes a null allele that has more severe phenotypes and characterizes the role of SAS-1 in various cell types and developmental stages within C. elegans (early vs. late development, formation of sensory neurons in larvae). They report a new localization pattern for SAS-1 at the base of the sensory neuron and demonstrate its requirement for chemosensation. They show that SAS-1 plays a role in recruiting another centriole protein, SSNA-1, both in C. elegans and when expressed in a heterologous system (U2OS cells).

Major concerns: none

Minor concerns:
1. Main Text: "Compatible with this view, AlphaFold2 multimer predictions suggest that SAS-1 interacts with SSNA-1 through its C-terminal alpha-helix (Fig. S5E, S5F), in line with structural evidence (Agostini et al. 2024)."
This statement is misleading, as Agostini do not report any evidence of SAS-1 interacting with SSNA-1. Also, without any follow-up experiments, I don't find this conclusion to be justified. AF2 will often pair up helices and give false predictions. To get some sense of the accuracy of this prediction, I would need to see the predicted aligned error plots, which the authors did not report here. I think this statement and Figure S5F should be removed.
> Apologies about this oversight -indeed, Agostini et al. 2024 do not provide evidence for SAS-1 and SSNA-1 interaction; this mention will be removed from the revised manuscript.

2. Figure 2B. The color for the "a/b tubulin" label should be magenta.
> We thank the reviewer for noticing this; the color label for "α/β tubulin" in Figure 2B will be changed to magenta.

3. Page 14, line 12. Check this sentence for errors.
> Thanks for noticing this typographical error, which will be corrected.

Significance
The study is well controlled, written, and conducted. The appropriate controls are used, and sample sizes and quantification are appropriate. The figures are clear and well-presented. Several studies have already examined a loss-of-function allele of sas-1, while the current study does similar analyses with a null allele and tissue-specific knockdown of SAS-1. The mechanistic and structural basis for how SAS-1 contributes to centriole integrity is limited. The most novel finding is that the centriolar protein SAS-1 is required for chemosensation in C. elegans. Thus, this study constitutes a small advance. This study will be of interest to cell biologists studying the regulation of centriole assembly and disassembly.

Reviewer #3
Evidence, reproducibility and clarity
Centrioles are thought to be extremely stable organelles, but there are multiple developmental contexts in which they are lost from cells. Building on previous work on the C2CD3 ortholog SAS-1 and centriole loss in C. elegans, here Jha et al. investigate the role of SAS-1 in maintaining centriole integrity and cilia function. Their work also reveals interactions between SAS-1 and SSNA-1 in centrioles and cilia. This study is well-done and an exciting advance for the field, as it further defines the mechanisms underlying centriole integrity and loss, as well as reveals new insight into cilia function. The major points in the paper are well-supported, and this manuscript should be published with revisions addressing the following major and minor comments.

Major comments:
1. The authors have generated a null allele of sas-1. The authors should compare the phenotypes of the null mutant to the hypomorphs. For example, are centrioles lost even more precociously in the germline compared to sas-1(t1521ts) (Pierron et al 2023)?
> We thank the reviewer for mentioning this important point. Accordingly, we will examine the temporal evolution of centriole loss during meiotic prophase in *sas-1(is13)* mutants compared to that occurring *sas-1(t1521ts)* mutants. See also point 1 of reviewer #1.

a. Related: In the U-Ex-STED images in Fig 1, in which region of the germline are the fragmenting centrioles found?
> The fact that these are nuclei from the mitotic region is spelled out in the figure legend.

2. In Pierron et al 2023, centriole widening occurred before centriole loss, and centrioles become prematurely wide in early prophase in the hypomorphic sas-1(t1521ts) mutant. These results suggest that centriole widening precedes centriole loss in C. elegans. However, in sas-1(is13), centrioles are narrower in the gonad (Fig S2J). Can the authors comment on how this result fits with the centriole integrity model? Does any change in centriole diameter mark the onset of centriole elimination?
> In the context of investigating the architecture and dynamics of centriole elimination during meiotic prophase in *sas-1(is13)* (see responses to point 1 of reviewer #1 and point 1 of reviewer #3), we will also address when centriole diameter becomes narrower, and expand on this finding accordingly in the revised manuscript.

3. The discovery that SAS-1 is involved in cilia structure and function is exciting, given that centrioles degenerate after ciliogenesis in C. elegans. Additional analyses of the role of SAS-1 and SSNA-1 in cilia would strengthen the manuscript.
> We will conduct additional experiments to address the questions of the reviewer, as summarized hereafter, thereby further clarifying the contributions of SAS-1 and SSNA-1 in cilium formation.
a. Does the central tube structure remain in cilia? Alternatively, is SAS-1 rearranged in cilia?
> Analysis by electron-microscopy revealed that the central tube is absent from cilia (PMID: 28411364).
b. Does SSNA-1 colocalize with SAS-1 at the transition zone?
> We will provide higher magnification views establishing such colocalization.
c. In the ZF::GFP::SAS-1 depletion, cilia are shorter. Which region of the cilium is affected - the proximal, middle or distal region?
> We assume that the reviewer refers to the ciliary base, the transition zone and the axoneme proper. We will assay markers specific for these regions to address this question.
d. Is this phenotype specific to the phasmid cilia?
> This question cannot be readily addressed, because the numerous amphid cilia are very close to one another, preventing robust measurements of individual cilia.
e. There is little to no effect on chemotaxis index in the ZF::GFP::SAS-1 treatment. The authors can try the dye-filling assay as an addition method to test cilia integrity
> We will conduct the suggested dye-filling assay.

Minor comment:
1. The authors should include description of meiotic stage when analyzing gonad regions (e.g. Fig 5E)
> We will indicate in the figure legend that these regions correspond to successive moments of meiotic prophase.

Significance

General Assessment:
The strengths of the study are in the careful analyses of mutant phenotypes, the excellent expansion microscopy imaging, and the novel result that SAS-1 and SSNA-1 are also involved in cilia structure and

function. The studies on cilia could be further developed to strengthen the manuscript.

Advance:
This work advances our understanding of centriole integrity, centriole composition, and cilia structure and function. It provides conceptual advances in our understanding of SAS-1 function and ciliogenesis in C. elegans.

Audience: This work will be of interest to cell biologists, in particular those with interests in centrosomes, cilia, and microtubules.

3. Description of the revisions that have already been incorporated in the transferred manuscript

4. Description of analyses that authors prefer not to carry out
