# Supplementary material for: C. elegans SAS-1 ensures centriole integrity and ciliary function, and operates with SSNA-1
Source: PLoS Genet. 2025 Oct 22;21(10):e1011912. doi: 10.1371/journal.pgen.1011912 (PMC12599939; doi:10.1371/journal.pgen.1011912)
Supplement: Attachment — Submitted filename: Response to reviewers.docx [file pgen.1011912.s013.docx]

**Manuscript number:** RC-2025-02998

**Corresponding author(s):** Pierre Gönczy

## 1. General Statements [optional]

We thank the three reviewers for their overall appreciation of our work, as well as for having asked us to resolve some points, including through additional experiments. We delineate hereafter how all the points that have been raised have been addressed, resulting in a markedly improved manuscript.

## 2. Description of the planned revisions

**Reviewer #1**

**Evidence, reproducibility and clarity**

SAS-1, a homolog to human C2CD3, is known to contribute to centriole integrity in C. elegans, though the molecular basis of this function has remained elusive. Here, Jha et al. generate the sas-1 null allele and employ U-Ex-STED microscopy to show that while SAS-1 is dispensable for centriole assembly, it is critical for maintaining organelle integrity. The authors also demonstrate that SAS-1 localizes to the transition zone of sensory neurons in L1 larvae. Using the ZIF-1-mediated tissue-specific degradation system in combination with an nphp-4 null mutant, a Y-link transition zone protein, they show that SAS-1 depletion exacerbates the posterior cilia shortening and chemotaxis defects observed in the null nphp-4 mutant, suggesting a partially redundant role for SAS-1 in ciliary function. Additionally, they demonstrate that SSNA-1, the C. elegans Sjögren Syndrome Nuclear Antigen 1 protein, localizes close to the SAS-1 C-terminus in the centriole architecture. SSNA-1 localization to the centriole during oogenesis and to the transition zone during ciliogenesis depends on SAS-1. Moreover, in the human co-expression system, SSNA-1 is recruited to the microtubule in a SAS-1-dependent manner. Collectively, these findings suggest that SAS-1 collaborates with SSNA-1 to maintain centriole integrity and support proper ciliary function.

Major Points
1. The title of the paragraph on Page 6, Line 2, "Loss of SAS-1 leads to premature centriole elimination during oogenesis," is misleading, as the authors do not present direct observation of centriole elimination in the germline. In wildtype, centriole elimination occurs from late pachytene to diplotene stage (PMID: 37987153). To support the current claim, it is necessary to examine SAS-4 and/or SAS-7 signals in the germline of control and sas-1(is13), to investigate whether the loss of SAS-1 leads to premature departure of SAS-4 and/or SAS-7 during meiotic prophase. Otherwise, I recommend rephrasing the title to more accurately reflect the data presented.

> We thank the reviewer for bringing up this important point. To address this issue, we conducted U-Ex-STED with SAS-4 and α/β-tubulin antibodies to assess centriole architecture across oogenesis (mitotic region, early prophase, late prophase) in control and *sas-1(is13)* mutants. As reported previously (Pierron et al., 2023), we found that in the control condition, centrioles widen in late prophase. In addition, as reported already in the initial submission, this analysis confirmed that centrioles are altered in the mitotic region of *sas-1*(*is13*) mutants, as one of the two centrioles exhibits a slightly smaller diameter whereas the other one is barely visible or absent altogether. Our new U-Ex-STED analysis reveals that these features persist as germ cell nuclei advance through oogenesis in *sas-1*(*is13*) mutants. Therefore, centrioles in *sas-1(is13)* indeed do not undergo stereotypic premature elimination. These new findings are reported in the figure panels Fig. S3A and S3B. The text of the manuscript, including the title of the paragraph, has been revised accordingly (p. 7 (l.2), 8 (l. 3-20)). See also major comment 1 of Reviewer 3.

In light of these findings, we propose that the second centriole -which is barely visible or absent altogether using U-Ex-STED -is the oldest one in each pair, which is known to serve as the principal site of PCM recruitment during mitosis. As a result, this older centriole may suffer damage from forces acting on spindle poles during mitotic division. By contrast, the procentriole would be less subjected to damage and remain comparatively intact until the next mitotic division. Furthermore, we hypothesize that those configurations in which the second centriole is absent correspond to a centriole that was made at least one cell cycle before the second centriole in those configurations where it is merely damaged. Once germ line nuclei enter the meiotic cell cycle, centrioles are no longer subject to mitotic forces, potentially explaining why this pattern remains in place until the onset of programmed elimination in diplotene. This explanation is provided in the revised Discussion section of the manuscript (p. 15 (l. 22-33), p.16 (l. 1-11)) and illustrated in two new Supplementary figure panels (Fig. S8A, S8B).

2. The localization of SAS-1 should be examined in Figure 1B-D and/or Figure 1G-I. Although protein expression of SAS-1 in wildtype and sas-1(is13) was assessed by Western blotting in Figure S1D, this analysis was performed on whole worm extracts. Therefore, it remains possible that residual SAS-1 protein is still present in the mitotic region, as the authors themselves note on page 7, lines 22-25. To address this concern, direct visualization of SAS-1 localization in the mitotic region of the gonad is necessary.

> We thank the reviewer for suggesting this critical control. We assessed the potential perdurance of maternally contributed TagRFP-T::SAS-1 by conducting live imaging of germline of *sas-1(is13)* homozygous mutants derived from heterozygous sas-1*(is13)/tagRFP-T::sas-1* mothers, finding no detectable maternally supplied TagRFP-T::SAS-1. Hence, no residual SAS-1 protein is present in the female germline or sperm of young adult *sas-1(is13)* homozygous mutants. This result is reported in the new panels Fig. S1F-H and in the text of the revised manuscript (p. 7 (l. 11-17)).

3. There is no explanation provided for the difference between Figures 2C and 2D, nor for the percentages shown in the top-right corners of the merged image in each condition.

>We apologize for this lack of clarity. The reported percentages reflect the fraction of sperm centriole pairs that fall into the two configuration types: one type with both centrioles intact (now Fig. 2C), and one type with one intact centriole and one ruptured centriole (now Fig. 2D). This is now clarified in the revised figure legend (p. 33 (l. 6-9)). Moreover, together with the findings in the female germline, we propose that the ruptured centriole corresponds to the older one, which was present at the onset of the first meiotic division, with those made thereafter being intact. This explanation is provided in the revised Discussion section of the manuscript (p. 16 (l.20-32)), and illustrated in two new Supplementary figure panels (Fig. S8C, S8D).

4. It is necessary to include SAS-7 localization in sperm centriole in Figure 2B-D, or alternatively, SAS-4 localization in Figure 2G-H. This inconsistent use of centriole markers makes it difficult to support the conclusion that the loss of SAS-1 allows sperm centrioles to be assembled but compromises their integrity, presumably leading to their rapid demise in the embryo following fertilization.

> Prompted by the reviewer’s suggestion, we conducted live imaging of embryos expressing SAS-4::GFP in both control and sas-1*(is13)* mutant conditions. Fully consistent with our initial findings with GFP::SAS-7, we observed that sperm-derived centrioles marked by SAS-4::GFP are rapidly lost following fertilization in sas-1*(is13)* mutants. These new results are reported in Figures 2G and 2H of the revised manuscript, and confirm that SAS-1 loss compromises the stability of sperm-contributed centrioles after fertilization. The initial GFP::SAS-7 data are now reported in Fig. S3F, S3G.

5. In Figure 4L-O, it is unclear how the authors defined the cilia length. For example, in Figure 4M, the distribution of CHE-11::mKate2 appears longer than the length indicated by the arrow. It would be helpful to provide a clear explanation of how cilia length was measured and to consider using an additional marker, such as OSM-6::GFP (PMID: 18316409), to more accurately define cilia length.

> We thank the reviewer for raising this important point. In the revised manuscript, we clarified how cilium length was measured, now by using SPY650-tubulin in strains expressing TagRFP-T::SPD-5 in four genetic backgrounds, measuring the distance from the TagRFP-T::SPD-5 signal to the tip of the SPY650-tubulin-marked axoneme. Using this more accurate approach, we found no significant difference in cilia length amongst genotypes. These new data are presented in the revised Figure 4G-K. In addition, we briefly explain the measurement method in the corresponding figure legend (p. 34 (l. 23-25)). Moreover, we restructured Figure 4 to incorporate these new results and improve clarity.

6. There is no direct evidence demonstrating that the C-terminus of SAS-1 binds to SSNA-1. In the human expression system shown in Figure 6, it would be helpful to test whether a C-terminal deletion of SAS-1 fails to recruit SSNA-1 to the microtubules. This would strengthen the conclusion regarding their interaction.

> As suggested by the reviewer, we tested whether a SAS-1 variant lacking the small region predicted by AlphaFold2 to interact with SSNA-1 (SAS-1ΔC, lacking amino acids 546-570), can recruit SSNA-1 to microtubules in U2-OS cells. Strikingly, we found that even though SAS-1ΔC localizes to microtubules like full-length SAS-1, it fails to recruit SSNA-1, further indicating that this region of SAS-1 is critical for interaction with SSNA-1. This important new finding is reported in Figure 6I and 6M, and mentioned in the accompanying text (p. 14 (l. 7-16)). See also minor point 1 of Reviewer 2.

Minor Points
1. In Figure 2A, the position of inset 3 at the 9 min timepoint appears misaligned relative to the corresponding DIC image.

> Thank you for noticing this misalignment, which has been corrected.

2. On page 10, line 34, the authors state that "ZIF-mediated depletion of SAS-1 from cilia results in a slight but significant chemosensation defect, which is enhanced in the nphp-4(tm925) background." However, in Figure 4R, no statistical test (e.g., t-tests) is shown comparing the control and Pdy-7::degron, and the data do not appear to show a clear difference between these two groups. I recommend rephrasing this conclusion.

> We have performed statistical analysis of the data shown in Figure 4R (Figure 4M in the updated manuscipt) and indeed found no significant difference in the chemotaxis index between control and SAS-1 depletion alone. We apologize for this oversight and have revised the corresponding text accordingly (p. 12 (l. 19-22)).

3. In Figure 5C, it is unclear why SAS-1 and SSNA-1 are examined at centrioles during meiotic prophase rather than in early embryos. The shift in experimental system without explanation makes this part of the manuscript difficult to interpret. It would be helpful to explain why meiotic prophase was chosen for this analysis.

> We chose to analyze nuclei in early meiotic prophase for U-Ex-STED because the relatively thin and well-spread gonad tissue allows for higher spatial resolution than what is possible in early embryos, where specimen thickness prevents high quality imaging. We mention this point explicitly in the revised manuscript (p. 13 (l. 6-9)).

4. It is unclear why sperm can assemble but then disassemble after fertilization (as shown in Figure 2B-H). Addressing this point in the discussion would help clarify the underlying mechanisms and better contextualize the findings.

> Prompted by the reviewer’s comment, we have expanded the Discussion to address this point. We now consider centriole stability more broadly and discuss how the absence of SAS-1 compromises centriole integrity over successive cell cycles. Within this framework, we also address why centrioles contributed by *sas-1(is13)* mutant sperm rapidly disassemble after fertilization (p. 16 (l. 28-32)), as well as new Supplementary figure panels Fig. S8A and S8B).

**Significance**

This manuscript presents significant and new mechanistic insights for the field, demonstrating that SAS-1 ensures centriole integrity and contributes to proper ciliary function through its collaboration with SSNA-1. These findings are supported by the precise generation of a sas-1 null mutant and the establishment of a neuron-specific degradation system. However, several conclusions appear overinterpreted, and the inconsistent use of centriole makers and tissue localization, as well as insufficient explanations of experimental design, make the manuscript difficult to interpret. Therefore, further validation is needed, along with clearer explanations of the experimental systems and quantification methods, as well as rephrasing, as described in major and minor points.

**Reviewer #2**

**Evidence, reproducibility and clarity**

This study investigates the role of the centriolar protein SAS-1 in centriole integrity, disassembly, and formation of functional sensory neurons. Previous studies already used a loss of function alleles for sas-1 to demonstrate that SAS-1 affects centriole disassembly and integrity in the gonad of C. elegans. This paper makes a null allele that has more severe phenotypes and characterizes the role of SAS-1 in various cell types and developmental stages within C. elegans (early vs. late development, formation of sensory neurons in larvae). They report a new localization pattern for SAS-1 at the base of the sensory neuron and demonstrate its requirement for chemosensation. They show that SAS-1 plays a role in recruiting another centriole protein, SSNA-1, both in C. elegans and when expressed in a heterologous system (U2OS cells).

Major concerns: none

Minor concerns:
1. Main Text: "Compatible with this view, AlphaFold2 multimer predictions suggest that SAS-1 interacts with SSNA-1 through its C-terminal alpha-helix (Fig. S5E, S5F), in line with structural evidence (Agostini et al. 2024)."
This statement is misleading, as Agostini do not report any evidence of SAS-1 interacting with SSNA-1. Also, without any follow-up experiments, I don't find this conclusion to be justified. AF2 will often pair up helices and give false predictions. To get some sense of the accuracy of this prediction, I would need to see the predicted aligned error plots, which the authors did not report here. I think this statement and Figure S5F should be removed.

> We thank the reviewer for pointing this out. Indeed, Agostini et al. 2024 do not report evidence for a direct interaction between SAS-1 and SSNA-1, and we have therefore removed this incorrect attribution. Pfister et al. 2025 mention such a plausible interaction by presenting an AlphaFold2 multimer prediction, but without any biochemical or functional validation. Importantly, by contrast, we now show experimentally that a C-terminal deletion of SAS-1 (SAS-1ΔC, aa 546–570) designed based on the AF2 predictions associates with microtubules like the wild-type protein but fails to recruit SSNA-1 (Fig. 6I, 6M). This provides the first functional evidence supporting the predicted SAS-1–SSNA-1 interface, thereby extending beyond the AF2-based hypothesis. See also main point 6 of Reviewer 1.

2. Figure 2B. The color for the "a/b tubulin" label should be magenta.

> We thank the reviewer for noticing this; the color label for "α/β tubulin" in Figure 2B has been changed to magenta.

3. Page 14, line 12. Check this sentence for errors.

> Apologies for the typographical errors in this sentence, which have been fixed in the revised manuscript (p. 17 (l. 5)).

**Significance**

The study is well controlled, written, and conducted. The appropriate controls are used, and sample sizes and quantification are appropriate. The figures are clear and well-presented. Several studies have already examined a loss-of-function allele of sas-1, while the current study does similar analyses with a null allele and tissue-specific knockdown of SAS-1. The mechanistic and structural basis for how SAS-1 contributes to centriole integrity is limited. The most novel finding is that the centriolar protein SAS-1 is required for chemosensation in C. elegans. Thus, this study constitutes a small advance. This study will be of interest to cell biologists studying the regulation of centriole assembly and disassembly.

**Reviewer #3**
**Evidence, reproducibility and clarity**
Centrioles are thought to be extremely stable organelles, but there are multiple developmental contexts in which they are lost from cells. Building on previous work on the C2CD3 ortholog SAS-1 and centriole loss in C. elegans, here Jha et al. investigate the role of SAS-1 in maintaining centriole integrity and cilia function. Their work also reveals interactions between SAS-1 and SSNA-1 in centrioles and cilia. This study is well-done and an exciting advance for the field, as it further defines the mechanisms underlying centriole integrity and loss, as well as reveals new insight into cilia function. The major points in the paper are well-supported, and this manuscript should be published with revisions addressing the following major and minor comments. 

Major comments: 
1. The authors have generated a null allele of sas-1. The authors should compare the phenotypes of the null mutant to the hypomorphs. For example, are centrioles lost even more precociously in the germline compared to sas-1(t1521ts) (Pierron et al 2023)? 
> We thank the reviewer for mentioning this important point, which has been addresed in full as delineated in response to major point 1 of reviewer #1.

a. Related: In the U-Ex-STED images in Fig 1, in which region of the germline are the fragmenting centrioles found?

> The fact that these are nuclei from the mitotic region is spelled out in the figure legend (p. 32 (l. 20)).

2. In Pierron et al 2023, centriole widening occurred before centriole loss, and centrioles become prematurely wide in early prophase in the hypomorphic sas-1(t1521ts) mutant. These results suggest that centriole widening precedes centriole loss in C. elegans. However, in sas-1(is13), centrioles are narrower in the gonad (Fig S2J). Can the authors comment on how this result fits with the centriole integrity model? Does any change in centriole diameter mark the onset of centriole elimination?

> We thank the reviewer for raising this interesting point. We conducted new experiments to measure centriole width in the mitotic zone, early prophase, and late prophase, and found that centrioles are consistently slightly narrower in sas-1(is13) mutants compared to controls in all three regions. This observation indicates that SAS-1 is required for achieving proper centriole diameter. Interestingly, a similar reduction in diameter has been reported for the sas-7(or452ts) mutant, in which the paddlewheel structure is absent (Sugioka et al., 2017). Consistent with this, we detect reduced GFP::SAS-7 levels in sas-1(is13) mutants (Fig. S2G–I), suggesting that impaired paddlewheel assembly may contribute to such narrower centrioles. In contrast to sas-1(t1521), where centrioles undergo premature widening before elimination (Pierron et al., 2023), this is not the case in sas-1(is13) animals. Together, these findings suggest that reduced centriole width in sas-1(is13) reflects a structural deficit linked to SAS-7/paddlewheel function rather than the onset of centriole elimination. We have included these notions in the revised Discussion (p. 16 (l. 12-17)). These new findings are reported in the figure panels Fig. S3A and S3B, with the text of the manuscript, included the title of the paragraph, revised accordingly (p. 7 (l.2), p. 8 (l. 3-20)). See also major point 1 of Reviewer 1.

3. The discovery that SAS-1 is involved in cilia structure and function is exciting, given that centrioles degenerate after ciliogenesis in C. elegans. Additional analyses of the role of SAS-1 and SSNA-1 in cilia would strengthen the manuscript.

> We have conducted additional analyses to further clarify the ciliary contributions of SAS-1 and SSNA-1, addressing the points raised the reviewer as follows:
a. Does the central tube structure remain in cilia? Alternatively, is SAS-1 rearranged in cilia?

> Prior analysis by electron-microscopy revealed that the central tube is absent from cilia. The relevant refrences are provided in the revised manuscript (Nechipurenko et al., 2017, Servas et al., 2018).

b. Does SSNA-1 colocalize with SAS-1 at the transition zone?

> In the revised manuscript, we provide high magnification views illustrating the colocalization between SAS-1 and SSNA-1 at the ciliary transition zone (new Figure S7C).
c. In the ZF::GFP::SAS-1 depletion, cilia are shorter. Which region of the cilium is affected - the proximal, middle or distal region?

> We thank the reviewer for this question. In the revised manuscript, we quantified cilium length using SPY650-tubulin (new Figure 4G-K). This analysis revealed no significant difference in cilia length upon ZF::GFP::SAS-1 depletion, either alone or in combination with *nphp-4(tm925).* As a result, we did not proceed to analyze specific ciliary region. See also major point 5 of Reviewer 1.
d. Is this phenotype specific to the phasmid cilia?

> Unfortunately, this question cannot be readily addressed for amphid cilia, which are numerous and very close to one another, preventing robust measurements of individual features.
e. There is little to no effect on chemotaxis index in the ZF::GFP::SAS-1 treatment. The authors can try the dye-filling assay as an addition method to test cilia integrity

> Following the suggestion of the reviewer, we performed dye-filling assays to test cilia integrity in phasmid neurons, finding a significant difference between control and SAS-1 depletion in the *nphp-4(tm925)* background. These findings are reported in the new Figure F4L and mentioned in the revised manuscript (p. 12 (l. 9-12)).

Minor comment: 
1. The authors should include description of meiotic stage when analyzing gonad regions (e.g. Fig 5E)

> Apologies for this oversight. We have indicated in the figure legend of the revised manuscript that the regions in Figure 5E correspond to successive moments of meiotic prophase of female germline maturation (p. 35 (l. 18)).


**Significance**

General Assessment: 
The strengths of the study are in the careful analyses of mutant phenotypes, the excellent expansion microscopy imaging, and the novel result that SAS-1 and SSNA-1 are also involved in cilia structure and function. The studies on cilia could be further developed to strengthen the manuscript. 

Advance: 
This work advances our understanding of centriole integrity, centriole composition, and cilia structure and function. It provides conceptual advances in our understanding of SAS-1 function and ciliogenesis in C. elegans. 

Audience: This work will be of interest to cell biologists, in particular those with interests in centrosomes, cilia, and microtubules.

## 3. Description of the revisions that have already been incorporated in the transferred manuscript

## 4. Description of analyses that authors prefer not to carry out
